# Random reward priming is task-contingent: the robustness of the 1-trial reward priming effect

**DOI:** 10.3389/fpsyg.2014.00309

**Published:** 2014-04-10

**Authors:** Árni G. Ásgeirsson, Árni Kristjánsson

**Affiliations:** ^1^Department of Psychology, Center for Visual Cognition, University of CopenhagenCopenhagen, Denmark; ^2^Laboratory for Visual Perception and Visuomotor Control, Faculty of Psychology, University of IcelandReykjavík, Iceland

**Keywords:** reward, visual attention, visual search, capture, repetition priming, visual selection, feature priming

## Abstract

Consistent financial reward of particular features influences the allocation of visual attention in many ways. More surprising are 1-trial reward priming effects on attention where reward schedules are random and reward on one trial influences attentional allocation on the next. Those findings are thought to reflect that rewarded features become more salient than unrewarded ones on the subsequent trial. Here we attempt to conceptually replicate this effect, testing its generalizability. In three versions of an analogous paradigm to the additional singleton paradigm involving singleton search for a Gabor patch of odd spatial frequency we found no evidence of reward priming, while we only partially replicate the reward priming in the exact original paradigm tested by Hickey and colleagues. The results cast doubt on the proposal that random reward enhances salience, suggested in the original papers, and highlight the need for a more nuanced account. In many other paradigms reward effects have been found to progress gradually, becoming stronger as they build up, and we argue that for robust reward priming, reward schedules need to be more consistent than in the original 1-trial reward priming paradigm.

## Introduction

Reward, financial or through the possibility of lessened effort, has a strong effect upon attentional function. Della-Libera and Chelazzi (2009) showed that selection or ignoring of stimuli is strongly modulated by whether stimuli are consistently associated with high or low reward. Anderson et al. (2011) found that stimuli associated with high reward are more likely to capture attention on subsequent unrewarded trials and in Kristjánsson et al. (2010) priming of pop-out was stronger for highly rewarded colors than colors receiving low reward. Kristjánsson et al. also showed that observers were flexible, quickly picking up on within-block changes in reward schedules even without awareness of the changes. Kiss et al. (2009) have shown how the N2pc attentional selection EEG component occurs earlier and is larger for visual search for colors consistently rewarded higher than for colors receiving low reward. Observer-by-observer N2pc correlated with effects of reward on search efficiency. Tseng and Lleras (2013) have then shown how rewarded search contexts are more easily learned during implicit contextual cueing (see Chelazzi et al., 2013 for review).

In most of these studies, reward was consistently associated with a particular color or context during a training phase, throughout testing, or at least for long series of adjacent trials. In contrast, Hickey et al. (2010a,b, 2011) reported an effect they called reward priming, in a task in which correct responses were rewarded with randomly determined high or low monetary reward. Their key finding was that a correct response to a target, which coincidentally resulted in high-reward, led to less attentional capture by an irrelevant singleton distractor on a subsequent trial when the color scheme of targets and distractors remained constant. If the colors changed between trials, on the other hand, performance was slowed. In one of their studies (Hickey et al., 2010b), low-magnitude rewards even led to an apparent devaluation of target features and attention was applied more slowly to these features. Participants responded slowly when the target was the same as on the preceding trial, but quickly when the colors swapped following low-magnitude rewards. Hickey et al. argued that rewards could have an inhibitory effect if target color differed from the last trial.

Hickey et al. termed this “reward priming”—the high reward biases selective processes toward a transiently valued stimulus feature, and can lead to inhibited responses if that feature is currently present on a distractor, analogously to priming of visual search (see e.g., Olivers and Humphreys, 2003; Muller et al., 2004; Theeuwes et al., 2006; Kristjánsson et al., 2008; Lamy et al., 2010; Ásgeirsson et al., 2014; see, e.g., Kristjánsson and Campana, 2010; Lamy and Kristjánsson, 2013, for review). What distinguishes this result from many other studies in the literature on reward and attention is that there was no contingency between target and distractor color on the one hand, and actual reward on the other. Hickey et al. seemingly isolated a direct effect of reward reception that was independent of longer-term, or motivational factors. In other words, this suggested 1-trial effects from rewarded attention deployments. Note that their effect was dependent upon the presence of an irrelevant color singleton, suggesting that the reward affects selection rather than other processes. Selection is, typically, much more difficult when a highly salient singleton distractor is presented alongside a less salient target stimulus (Jonides and Yantis, 1988; Theeuwes, 1992; Franconeri et al., 2005). That reward priming affects efficiency of selection is further supported by electrophysiological evidence. High rewards affect the N2pc ERP component differentially; depending on whether the color scheme of a display repeats or the colors swap (Hickey et al., 2010b).

Hickey et al. proposed that this reflected a general transient effect of reward associated with a particular target color that boosts target saliency. Throughout their experiments, they always used the same stimulus set, with set-size, color, and target identity manipulations; never manipulating salience directly or generalizing the effects to other stimulus sets [but see Hickey and van Zoest (2012) for related findings with eye-movement measures]. Here we conduct a conceptual replication of their experiments to generalize their findings to other stimulus sets (Experiments 1 and 2).

Another point that merits attention is that the main dependent variable in the tasks of Hickey et al. is response time. Since reward did not depend on response speed but only on whether the response was correct or not, this means that the most lucrative strategy was not to respond as quickly as possible, but rather to respond correctly. The main dependent measure is, in other words, not rewarded. A second motivation here was therefore to investigate the role of strategy, by adding motivation for speed (Experiment 3), where observers were told that they had 45 min to maximize their earnings. The faster they responded the more trials were presented so that their chances to earn money increased. Finally, in experiment 4 we attempt to replicate the Hickey et al. result using their original task.

## Experiment 1—conceptual replication of Hickey et al. (2010a)

**Figure 1:**
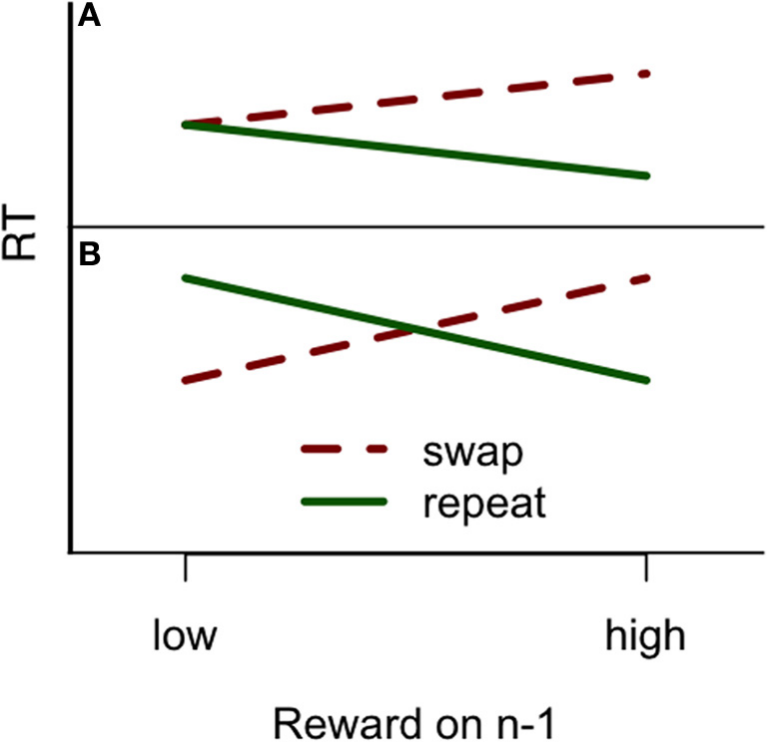
**(A)** The reward priming pattern reported in Hickey et al. (2010a), where low reward did not affect the subsequent trial, but a high reward did so contingent on whether color was repeated or not. **(B)** The reward priming pattern reported in Hickey et al. (2010b, 2011), where both high and low reward affected response times, contingent on color repetition, but in the opposite ways, as if a target previously associated with high reward was subsequently highly valued, but a target previously associated with low reward was subsequently devalued. The figure does not represent actual data and is only for illustrative purposes. See Hickey et al. (2010a,b, 2011) for details on their results.

The first experiment was designed to conceptually replicate the results of Hickey et al. (2010a) testing a task similar to the one used in their experiments, while changing the stimuli in the hope of generalizing the results beyond their exact paradigm. If reward priming reflects increased salience of recently rewarded features, the effect should be replicable in any paradigm where there is sufficient selection pressure, and the random feature (e.g., color) is sufficiently salient. We therefore expected to find results consistent with 1-trial reward priming in our conceptually identical paradigm. From the Hickey et al. (2010a) result we expected observers to respond faster to a target whose color is repeated from the previous trial if it also resulted in a high reward. However, if the target changes color between trials and observers have previously received high reward, they should respond more slowly to the current target. Such a divergence in results would constitute reward priming following high, but not low reward but no color-association effects following low reward trials (Figure 1A). But based on Hickey et al. (2010b, 2011) we might also expect the opposite pattern to the high reward pattern for trials following low reward—that observers will respond slowly to targets sharing color with a previous target. Conversely, observers should be faster when a target changes color between trials, immediately following reception of low reward (Figure 1B). Tentatively, we will consider the emergence of either pattern a successful replication.

### Methods

#### Subjects

Twenty observers participated. They were randomly assigned to either experiment 1 or experiment 3 from a pool of 40 participants (26 female), aged 19–30 year (mean = 23.8 year). Due to privacy restrictions, age and gender information is not available for each experiment separately. The project was approved by the Research Ethics Committee of the Department of Psychology, University of Copenhagen, and the IRB at the University of Iceland.

#### Stimuli and apparatus

**Figure 2:**
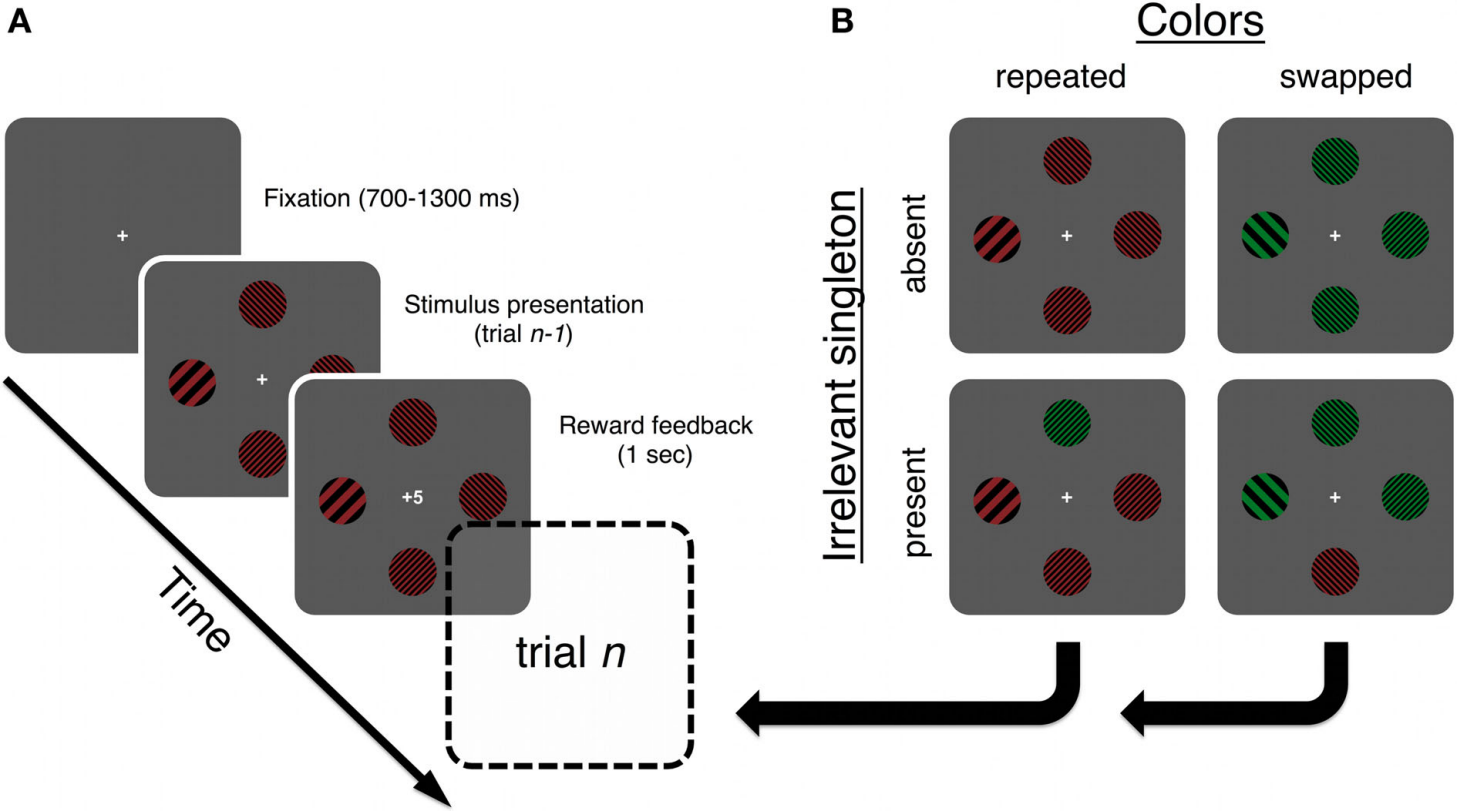
**(A)** A trial started when a fixation-cross appeared. The stimulus array was presented until response, followed by a feedback display signifying the amount of reward or punishment obtained on the current trial. **(B)** All possible trial types relative to the example trial type in panel **(A)**. Each type was equally likely and the amount of reward was not related to trial type or any other task attribute. Note that the main hypothesis is tested by calculating the means of trials where the irrelevant singleton was present on the current trial, but absent on the previous (n-1) trial. The target is always shown as a low spatial frequency singleton in the western position. Targets could also be high spatial frequency singletons, among low-frequency ones, and were equally likely to appear at any of the four positions. Stimuli are not drawn to scale. See Stimuli and apparatus for stimulus specifications.

***Stimuli***. Gabor-patches of low or high spatial frequency (approx.1 and 4 cycles per degree, respectively), appeared on a dark background (lum. = 0.2 cd/m2). The diameter of each Gabor was 4.3°. They were tilted ± 45° from vertical (see Figure 2). The target and distractor stimuli were pinkish red (*x* = 0.412, *z* = 0.304, lum. = 35.6 cd/m2, at maximal luminosity) or light green (*x* = 0.288, *z* = 0.384, lum. = 32.1 cd/m2, at max. luminosity). On each trial, four Gabor patches were presented on an imaginary circle (radius = 7.4°), centered on a fixation cross. Stimulus configuration varied randomly between trials such that the distance between all 4 stimuli was always equal, but there were 12 potential stimulus positions, resulting in three different configurations. A target was defined as the stimulus with the odd spatial frequency, whereas all other stimuli were of the opposing spatial frequency.

A target, defined by odd spatial frequency, was present on all trials. A salient singleton distractor was present on 50% of trials, while the other half had only non-targets plus the target. We will refer to these as present/absent trials throughout, unless otherwise noted. On present trials, there were always two non-targets on the screen, alongside the target and the singleton distractor. The non-targets always shared the color of the target, but shared spatial frequency with the oddly colored singleton distractor. On absent trials, there were three non-targets while all other features were the same as on present trials.

The percentage of present vs. absent trials differed from Hickey et al. (2010b), where a singleton distractor was present on 80% of trials. The reasoning behind this change is that Hickey et al. (2010a, p. 4) argued that “novelty” should disrupt search to the greatest degree, and therefore based their analysis solely on trials where a singleton distractor was present, but had not been present on the previous trial^1^. We therefore reasoned that disruption by novelty would increase with more absent trials. Simultaneously, we increased the statistical power of our design, by keeping 25% of our trials eligible for analysis (half of our trials are present trials, and half of those will follow an absent trial), compared to 16% in Hickey et al. (2010b; 80% of their trials were present trials, but only 20% of those followed an absent trial).

***Reward schedule***. As in the studies by Hickey et al. (2010a,b, 2011), the reward schedule was not contingent on any display parameters, but selected by a balanced randomization algorithm for each trial (“high” reward = (5 ISK/0.3 DKK); “low” reward = (0.5 ISK/0.03 DKK). Punishment (following errors) had only one level, equal to the negative of “high” reward. Rewards were also signaled by audible feedback. A high-pitched “ka-ching” sound was played following high reward; when the reward was low, a high note was played (C6); and when the response was incorrect and the observer was punished, a medium pitched note was played (C4). Examples of these feedback noises were given before the experiment started.

#### Apparatus

The experiment was carried out in two laboratories and apart from any differences noted, methods were identical. At the University of Iceland, the experiment was run on a 2.8 GHz Dell Optiplex 760 desktop computer connected to a 100 Hz 14′ CRT display. At the University of Copenhagen, it was run on a 2.66 GHz Dell Optiplex 255 connected to a 100 Hz 19′ CRT display. Stimulus presentation was programmed in Matlab® using the Psychophysics Toolbox (Brainard, 1997; Pelli, 1997). Viewing distance was adjusted so that retinal size was practically identical in the two setups.

#### Design and procedure

Observers were presented with illustrated task instructions before the experiment started. They were instructed to respond as quickly as possible, without making many errors. The reward scheme was explained, i.e., that when responding correctly observers would receive high or low reward, but when they responded incorrectly they would lose money. Following the instructions they viewed and listened to computerized examples of “dummy” trials and the audio feedback related to each reward/punishment level, before completing 30 practice trials. During practice trials, reward balance was displayed (but not paid out). Observers were informed of this beforehand. Following practice the experiment started, run as a single block of 900 trials. Four observers ran only 811 trials, but were otherwise treated identically to all other observers.

A single trial started with the presentation of a fixation cross at screen center for 700–1300 ms after which the four Gabor patches were presented on an imaginary circle. On half of trials, the Gabor patches were all uniformly colored, red, or green (singleton distractor absent trials). On these trials, there were always three non-targets whose spatial frequencies were the same and one target, defined by odd spatial frequency. On the other half of trials, three Gabor patches, two of which were non-targets and one a target, shared a color, while the fourth patch had the opposing color (the singleton distractor). Note, however, that stimulus colors were completely irrelevant to the task.

Observers located the item of odd spatial frequency (the target-defining variable) and reported whether that Gabor was rotated −45° (“J” key) or + 45 (“L” key) from vertical (the response feature). As soon as a key was pressed, the reward amount appeared at screen center and the appropriate feedback sound was played. The next trial started a second later with the presentation of the fixation cross in isolation. This procedure was interrupted every 30 trials by the presentation of the total amount earned. After finishing the experiment, observers were debriefed on the experimental hypothesis and informed of their total earnings.

### Results

Before analyses, we applied individual filters to each observer's data where very slow reaction times were discarded (individual mean + 3 std; 1.8% of trials, between-observer range: 0.2–2.6%). As expected there were large differences between trials where the irrelevant singleton distractor was present vs. absent [810 vs. 699 ms, respectively; *t*_(19)_ = 8.409, *p* < 0.001]. This shows that our modified irrelevant singleton paradigm is analogous to the one in Theeuwes (1992), in that observers have trouble ignoring color singletons, even when always irrelevant. Observers were also more accurate in the distractor absent condition (98.5%) compared to the distractor present condition [97.2%; *t*_(19)_ = −3.337, *p* < 0.004].

We then filtered the dataset further to match that of Hickey et al. (2010a, p. 4). We discarded all incorrect trials, as well as trials preceded by incorrect trials since they were also not preceded by reward. We also discarded singleton distractor absent trials, and trials that were not preceded by absent trials (see also Method). Finally, we limited analyses to trials where response/orientation was repeated from the previous trial. The filtering process left an average of 101 trials per participant.

**Figure 3:**
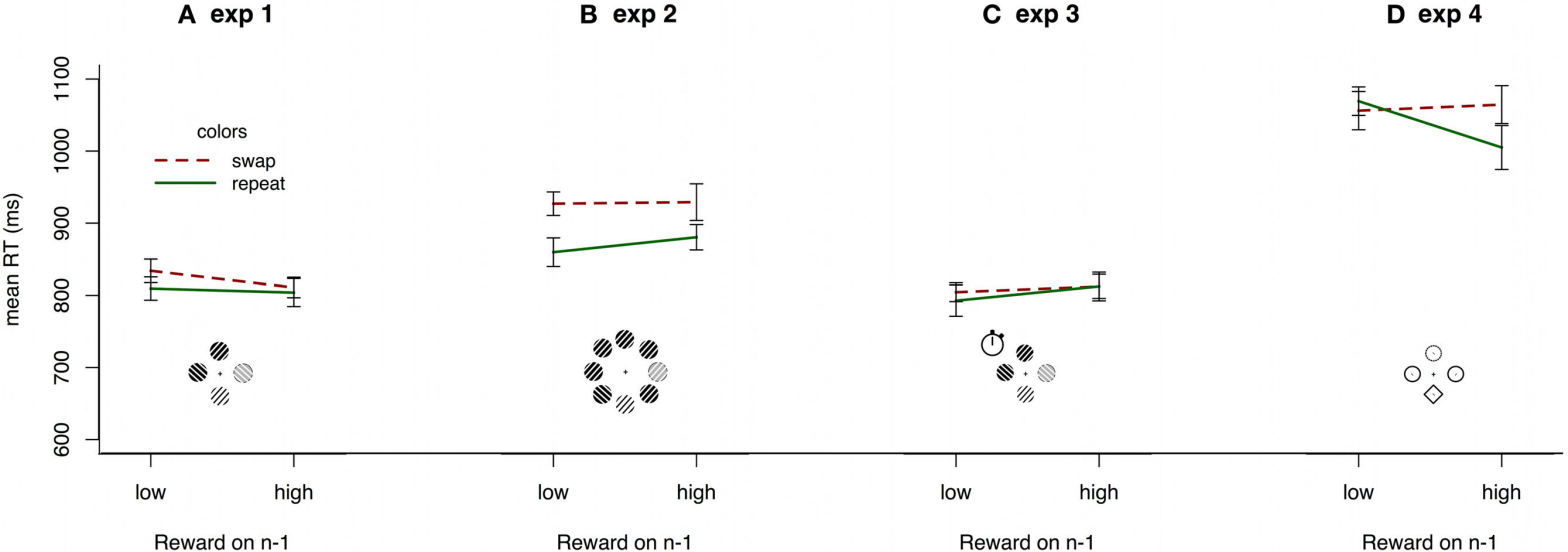
**Mean reaction times in experiments 1-4, for all observers by immediately preceding reward and repetition or switch of the color scheme from the preceding trial**. Search arrays show stimuli and set-sizes in each experiment (not drawn to scale; see Methods for details). Error bars show within-subject 95% confidence intervals (Cousineau, 2005).

Following Hickey et al. (2010a) we contrasted reaction times where target color is constant between subsequent trials compared to when it changes and, further, whether the current trial is preceded by randomly determined high or low reward. A repeated measures ANOVA of within-subject effects on reaction times revealed a marginally significant main effect of target color repetition [*F*_(1, 19)_ = 4.236, *p* = 0.054, η^2^_*p*_ = 0.182] but no effect of reward value [*F*_(1, 19)_ = 1.129, *p* = 0.301, η^2^_*p*_ = 0.056]. Most importantly, there was no interaction between target color repetition and reward value [*F*_(1, 19)_ = 1.612, *p* = 0.220, η^2^_*p*_ = 0.078]. Figure 3A, shows no hint of the reward priming interaction of reward and color reported in Hickey et al. (2010a). Taking accuracy into account, by calculating inverse efficiencies (e.g., Townsend and Ashby, 1983) for each condition and running a repeated measures ANOVA on those, further pushed the trend away from the reward priming interaction.

### Discussion

There was no evidence of reward priming in experiment 1, or faster responding following highly rewarded trials, when target color was constant between trials, nor a slowing of responses following high reward with a swap of colors. In fact reward barely seemed to affect performance at all. This is surprising, given that Hickey et al. (2011) argued that the salience of the target feature is boosted following a highly rewarded trial, and observed the effect on three separate occasions in a paradigm analogous to ours.

Two features of the respective paradigms should be noted: (i) Our set-size was fixed at 4. Hickey et al. demonstrated reward priming using set-sizes of 6 and 12. Might set-size affect the results? When target identity is unknown in visual search, slopes of set-size vs. response time are negative, decreasing toward an asymptote around a set-size of 10 (Bravo and Nakayama, 1992). This has been attributed to ambiguity (Meeter and Olivers, 2006); with only two non-targets and an unknown target, there is sparse information to determine the target. However, when a target is present among many identical non-targets, there is ample evidence regarding which category each stimulus belongs to. Reward priming may therefore depend on sufficiently unambiguous search conditions. In experiment 1, we used the smallest possible set-size, and consequently the most ambiguous target-distractor relationship, in an irrelevant singleton paradigm, finding no reward priming.

(ii) An important feature of Hickey et al.'s paradigm is that reward value is unrelated to reaction time. If an accurate response is given, reward level is random. The optimal strategy to maximize profit is therefore to emphasize accuracy using a conservative (i.e., slow) response strategy. The adoption of such a strategy would compromise the interpretation of reaction times, the primary measure.

We address these two points in experiments 2 and 3.

## Experiment 2—increased set-size

May set-size explain discrepancies between our results and those of Hickey et al.? They originally used a set-size of 12 (2010a,b) and replicated the effect with a set-size of 6. The reward priming effect may depend on larger set-sizes, perhaps reflecting larger pop-out for the target and irrelevant distractor (see Bravo and Nakayama, 1992; Meeter and Olivers, 2006). We therefore re-ran experiment 1 on 19 naive observers, doubling the number of objects in each search display.

### Methods

Nineteen observers (aged 18–38, mean 24.3 years; 15 female) participated in this experiment and experiment 4. The order of the experiments was counterbalanced so that 10 observers ran experiment 4 first, followed by experiment 2, but the remaining 9 in reverse order. Methods were identical to experiment 1, except for increased set-size (8). Stimuli were now presented at 8 fixed positions on an imaginary circle. The size of each Gabor-patch was reduced slightly to fit well on the screen (from 3.71 to 3.4°), proportionally decreasing spatial frequency. Apparati were identical to experiment 1 (Copenhagen setup).

#### Personality trait measures

Hickey et al. (2010a) showed that reaction time reductions immediately following reception of high reward correlated strongly with a subscale on the BIS/BAS Scale measuring sensitivity to punishment and reward (Carver and White, 1994). However, the correlation did not hold for the opposite negative priming effect, following low reward reception. We administered the BIS/BAS scale to the 19 observers. Response sheets were signed with observer numbers and returned in closed envelopes to ensure anonymity. Results from trait measures are presented at the end of the results section for all experiments.

### Results

Observers responded accurately on 98.4% of trials in experiment 2 (range: 95.9–99.8). The dataset was filtered in exactly the same way as in experiment 1, leaving an average of 106 trials per observer. Again, there were clear differences between reaction times in the present (905 ms) and absent (744 ms) conditions [*t*_(18)_ = 7.572, *p* < 0.001]. A repeated measures ANOVA revealed a main effect of color repetition [*F*_(1, 18)_ = 13.097, *p* = 0.002, η^2^_*p*_ = 0.421] but no effect of reward [*F*_(1, 18)_ = 1.726, *p* = 0.205, η^2^_*p*_ = 0.087]. Most importantly, there was no indication of the reward priming interaction [*F*_(1, 18)_ = 0.981, *p* = 0.335, η^2^_*p*_ = 0.052; Figure 3B].

### Discussion

With increased set size, to induce larger pop-out effects (Bravo and Nakayama, 1992) there was still no evidence of feature-specific reward priming. We can therefore rule set size out as a potential explanation of the results of experiment 1. We are, however still left with the puzzle of not observing any reward priming, which is clearly predicted by Hickey et al, who claimed that reward boosts the saliency of a rewarded feature on the next trial.

## Experiment 3—encouraging speeded responding

The main dependent measure in Hickey et al. (2010a,b, 2011) was RT, but maximizing speed was not the optimal strategy. Monetary gains were highest by emphasizing accuracy over speed, since errors were punished by losses. Even if Hickey et al. found reward priming with this procedure; this might explain the null findings in our first two experiments. Subtleties in instructions may have motivated observers to respond quickly and accurately in their studies, while our instructions failed to do so. However, an instruction manipulation, with constant task contingencies, would not ensure that observers would adopt a faster, less optimal, strategy. Instead, in experiment 3, we changed the task such that, rather than each observer participating in 900 trials exactly, observers had 45 min to perform as many trials as possible. This manipulation should motivate observers to respond quickly and accurately, and discourage conservative response strategies.

### Methods

Twenty observers participated (see Method, Experiment 1 for details). The results from one observer were discarded due to poor performance (accuracy < 80%; mean RTs > 2000 ms). Other features of the design were copied directly from experiment 1, with the exception that time, rather than trial number now limited experiment duration. Whereas experiment 1 was run for a predefined number of trials, observers were told that they had exactly 45 min to finish as many trials as possible. Instructions were changed to highlight this, along with encouragement that they should try to earn as much money as they could in that time. The BIS/BAS scale was administered before observers performed the primary task with identities protected as in experiment 2.

### Results

Observers finished 925 trials during their 45 min on average (range: 791–987). As before, there were clear differences between reaction times in the distractor-present (794 ms) and distractor-absent (703 ms) conditions [*t*_(18)_ = 8.333, *p* < 0.001]. There was no difference in overall mean reaction time between this task and experiment 1 [747 vs. 753 ms, respectively; *t*_(34.971)_ = 0.151; *p* = 0.881]^2^. The similarity of mean reaction time between the two versions of the task may suggest that lack of motivation was not a key factor in producing the null-effect in experiment 1. We cannot rule out that the group participating in the current experiment was slower in general (nor can we rule out the opposite), since these were different observers. Further, the similarity in overall reaction times says nothing about whether there are effects of the limited time motivation in conditions crucial for reward priming.

To test whether the motivation manipulation produced reward priming, we filtered the data in the same way as in experiment 1, leaving an average of 109 trials per observer. A repeated measures ANOVA of within-subject effects on reaction times revealed no significant main effects of color repetition [*F*_(1, 18)_ = 0.199, *p* = 0.661, η^2^_*p*_ = 0.011], reward value [*F*_(1, 18)_ = 3.136, *p* = 0.093, η^2^_*p*_ = 0.148], nor an interaction between the two [*F*_(1, 18)_ = 0.307, *p* = 0587, η^2^_*p*_ = 0.017]. Figure 3C clearly shows that there is no trend toward any reward priming interaction. In fact, the only trend in the data is a non-significant negative effect of preceding reward value, the opposite of reward priming. As in experiment 1, inverse efficiency analysis did not reveal any effects (*F*'s < 1, *p*'s > 0.443).

### Discussion

Although we incentivized faster responding, we found no hints of reward priming in experiment 3. All our efforts to replicate Hickey et al. in a paradigm conceptually identical to theirs have failed. The most sensible reason left is that superficial differences between our paradigm and theirs explain the discrepancy. The reward priming effect may be specific to tasks with certain minute attributes. In experiment 4, we tested whether we could produce reward priming using the original paradigm by Hickey et al. (2010a,b).

## Experiment 4—direct replication of the reward priming paradigm

In experiment 4, we used the same stimulus set as Hickey et al. but kept the set-size of 4 and the same instructions as in experiment 1, to try to replicate their reward priming effect.

### Methods

**Figure 4:**
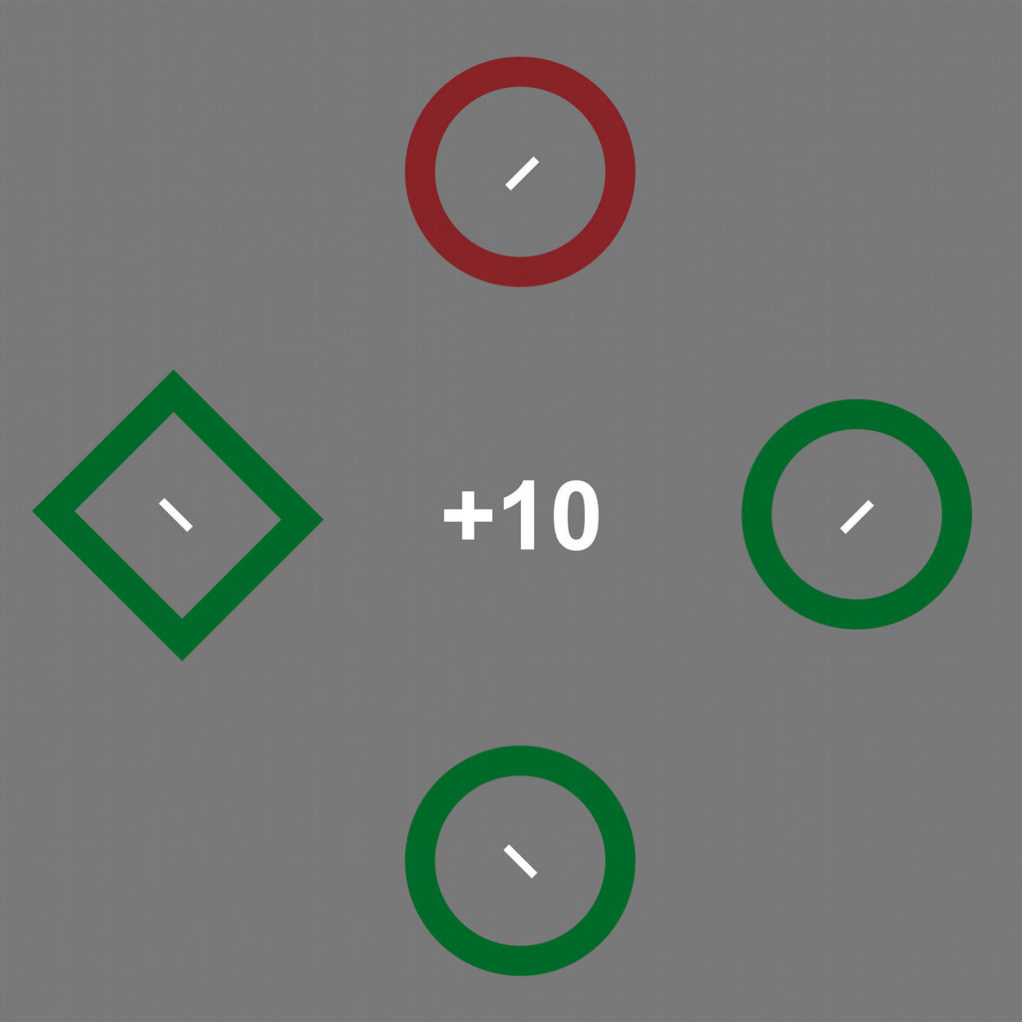
**An illustration of the stimuli used in experiment 4**. Observers were to locate the target with the odd shape (the green diamond) and report the tilt of the line inside it. The example in the figure shows a trial with an irrelevant singleton (the red circle). Stimuli are not drawn to scale (see Method for details).

Observers were the same as in experiment 2. The stimuli were approximate copies of those used by Hickey et al. (2010a). Targets were defined by shape, rather than spatial frequency and could be either diamonds or circles. A circular stimulus had a diameter of 4.3° while the diamonds were tilted squares (by 45°) with sides of 3.7° visual angle. The stimuli were either red (*x* = 0.580, *z* = 0.322) or green (*x* = 0.296, *z* = 0.529) and approximately equiluminant at 31 cd/m2. As in the original additional distractor reward paradigm (e.g., Hickey et al., 2010a), the response feature was a small bar (0.8° long, <0.1° wide) at the center of each search stimulus, but they were tilted by ±45°, to make the response demands identical to experiments 1–3 (Figure 4). This is in contrast to the horizontal/vertical distinction in the original paradigm. Other aspects of the methods and procedure were identical to experiment 1.

### Results

The data were filtered in the same way as previously. A repeated measures ANOVA revealed no main effect of color repetition [*F*_(1, 18)_ = 1.844, *p* = 0.191, η^2^_*p*_ = 0.093], but showed a main effect of reward value [*F*_(1, 18)_ = 5.78, *p* = 0.027, η^2^_*p*_ = 0.243]. Most importantly, for the first time here, there was a marginally significant interaction between color repetition and reward value [*F*_(1, 18)_ = 4.711, *p* = 0.044, η^2^_*p*_ = 0.207] matching the qualitative pattern of reward priming (Figure 3D). There is therefore a small reward priming effect, dependent on color repetition following high reward trials. However, although there is a small trend toward an inhibitory effect when colors swapped following high reward, this was far from significant [*t*_(18)_ = 0.400, *p* = 0.694].

### Discussion

In experiment 4, we only partly replicate the results of Hickey et al. (2010a,b, 2011). Following high reward, search was speeded if search colors were identical but not if they switched. If the reward was low there was no effect of the color scheme on the preceding trial. We are, however, still left with the puzzle that in a conceptually identical paradigm tested three times (Experiments 1 to 3) this reward priming from non-feature-contingent reward is not observed. Hickey et al. argue that “reward acts largely to prime target representations, consistent with the idea that objects associated with good outcome become visually salient” (2011, p 117). According to our results this conclusion applies, at best, to a subset of stimulus types, laying an important constraint upon the generalizability of reward priming. Reward has a strong effect on attentional function in various other paradigms, but the reward schedules in these paradigms were not random as here (Della-Libera and Chelazzi, 2009; Kiss et al., 2009; Kristjánsson et al., 2010; Anderson et al., 2011). Results of future studies will have to determine how general the non-contingent reward priming effect is.

## Individual differences in experiments 1 to 4

**Figure 5:**
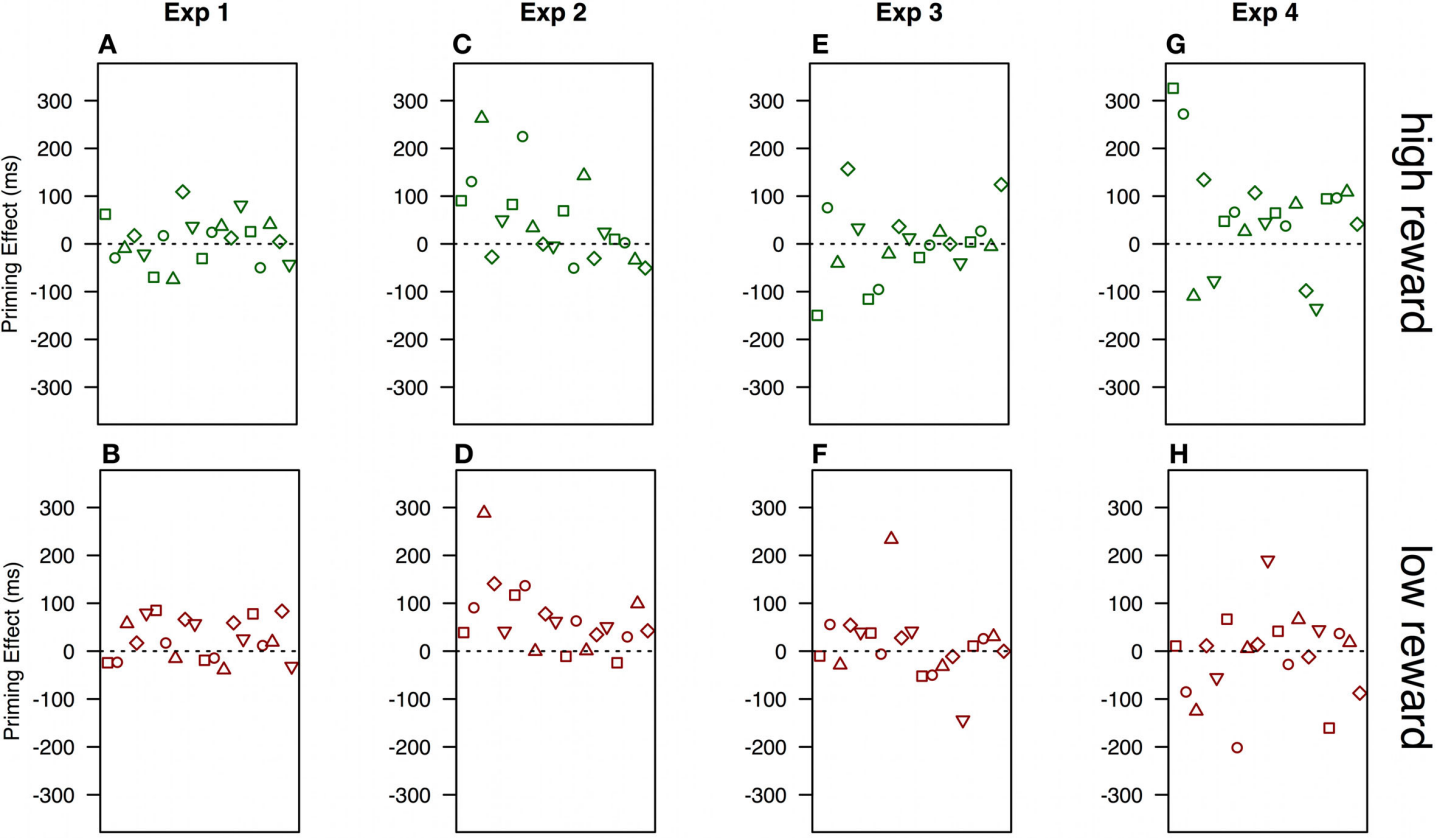
**Differences between repeated vs. swapped color displays (RT_swap_ - RT_repeated_) when previous reward was high (top panels) and low (bottom panels)**. Each point represents one observer. The order of observers is maintained within each experiment. Therefore, a single observer's priming effects following high and low reward can be identified by order and data-point symbols. The symbols have no significance besides easing within-subject comparisons.

There are marked differences in data patterns of individual observers, and from that point-of-view, any systematic patterns are hard to discern. Figure 5A shows the variance in priming effects, mean RT_swap_-mean RT_repetition_ in experiment 1 when previous reward was high. Positive priming effects were observed for only 12 out of the 20 observers, and in contrast to Hickey et al. (2010b) most of the observers also show positive priming effects in the low reward condition (Figure 5B). A similar pattern, only stronger, appears in experiment 2 (set-size = 8), where a large majority of observers show positive color priming effects under both reward conditions (Figures 5C,D). In experiment 3, observers were approximately equally likely to show positive and negative priming effects in both reward conditions (Figures 5E,F). Only in experiment 4, where we used the original stimulus set (e.g., Hickey et al., 2010a), was there any divergence of data patterns following high and low reward. Here, 15 out of 19 observers showed positive priming effects following high reward (Figure 5G), while the effects following low reward were scattered almost equally around 0 (Figure 5H).

In Table 1 we show correlations between priming effects on trial *n* conditional on reward reception on trial *n-1* and BIS/BAS subscale scores (Carver and White, 1994). We were particularly interested in the BAS_drive_ subscale because Hickey et al. (2010a) found a strong positive correlation between this subscale and priming effects following highly rewarded trials. BAS_drive_ measures persistence in achieving a desired goal, i.e., reward seeking. The BAS_reward responsiveness_ subscale is also of interest since it measures sensitivity to the occurrence or reception of reward.

**Table 1:**
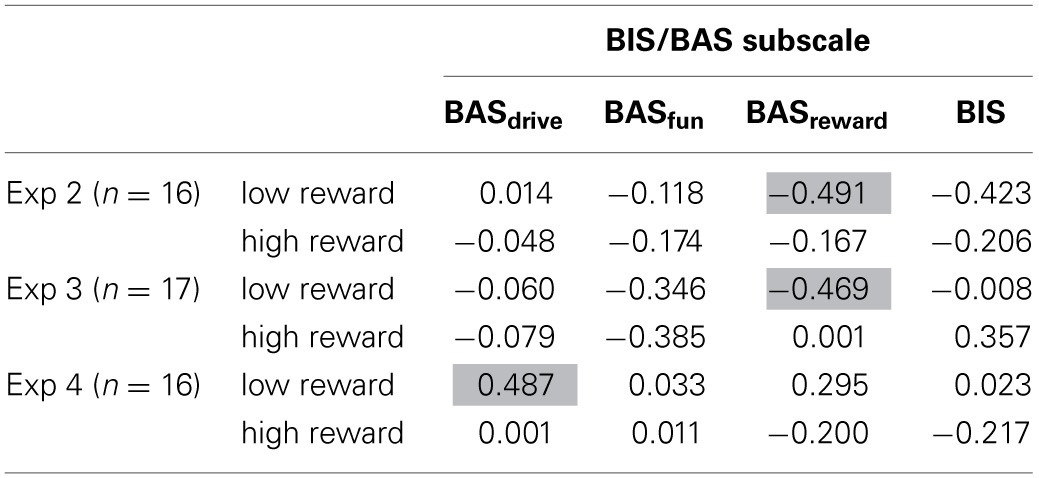
**Correlation coefficients (Pearson's *r*) between subscales of the BIS/BAS scale and priming effects under high and low reward conditions**.

*No correlations were significant. Highlighted cells indicate marginally significant p-values (<06)*.

Sixteen out of 19 observers in experiments 2 and 4 responded to the BIS/BAS scale. Two observers chose not to respond, or did not respond to all items. In experiment 3, 17 out of 20 observers responded to the full questionnaire. Table 1 shows correlations between the subscales of the BIS/BAS scale and priming effects following low and high reward trials. No correlations were significant, an unsurprising result with so few respondents, but three correlations were marginally significant (*p* < 0.06). It is interesting to note that, in experiment 4, where we used the stimulus-set of Hickey et al. (2010a,b), there is no indication of a relationship between BAS_drive_ and high reward. However, there are hints of a positive relationship between BAS_drive_ and low reward reception, i.e., that those more persistent in seeking out positive reward outcomes are more likely to be positively primed by the low reward-color association. There may also be relationships between observers' sensitivity to reward reception (BAS_reward responsiveness_). In experiments 2 and 3 there is a negative correlation (Table 1) between priming effects following low reward reception and reward responsiveness, i.e., those most positively affected by reward reception are most likely to devalue a color, following a co-occurrence with low reward. Notably, the three strongest correlations are all related to low reward reception. Note, however that the psychophysical data shows no low-reward and color associations in any of the 4 experiments (Figure 3), meaning that these correlations reflect the large between-subject variances in performance, but not systematic population effects.

## The carrier of the reward priming effect

A last attempt at explaining the discrepancies between experiments 1–3 and experiment 4 (see also Hickey et al., 2010a,b), relates to the potential carrier of the reward priming effect. In visual search, carriers of different effects can change depending on task-demands and/or minute stimulus attributes. An example is the attentional repetition priming effect that, under certain conditions, is purely feature based (i.e., Kristjánsson, 2006, 2009; Ásgeirsson et al., 2014) whereas a small change in feature configuration can facilitate object—rather than feature—based priming (Kristjánsson et al., 2008, see also Ásgeirsson and Kristjánsson, 2011). It is possible that for reward priming, the whole object must be repeated, i.e., color, spatial frequency and orientation, and that this might account for the absence of reward priming in experiments 1 to 3, although this is unlikely in light of the results of Kristjánsson(2006), (see Kristjánsson and Campana, 2010 for review). After all, the lines to be judged in Hickey et al's experiments were not connected to the colored items that either repeated or changed. We therefore, re-ran our analyses from experiments 1–3 with an additional filter. Only trials where spatial frequency was repeated (along with earlier filters, see Method, Experiment 1) were analyzed.

If our paradigm results in reward priming, but objects, rather than features carry the effect, we should see the qualitative pattern of Hickey et al. (2010a) if we analyze only trials where a full object is repeated from the preceding trial. Repeated measures ANOVAs on data filtered in this new way did not reveal any interactions between reward and color repetitions (*p*'s > 0.218), nor any hint of object-based reward priming at all in experiments 1–3.

## Controlling for other variables

Another potential issue that needs addressing is whether the two different spatial frequencies affect the processing of the stimuli, and could therefore disguise reward priming effects. In principle, reward priming could be present for only one category of spatial frequencies. Therefore, we re-ran the analyses from experiments 1–3 with the addition of spatial frequency as an independent factor in repeated measures ANOVAs. In all three experiments, we confirmed main effects of spatial frequency (*F*'s ranged 17.3–31.4; *p*'s < 0.001), where observers responded faster to the low frequency targets (range: 54–82 ms). However, spatial frequency never interacted with reward (*p*'s > 0.361), color repetition (*p*'s > 0.108) or the combination of all three factors (*p*'s > 0.214). Based on these analyses, we cannot conclude that the current results are explained by the difference in defining variable characteristics (i.e., Spatial frequency).

As mentioned in the *method* section for experiment 1, the data in different publications from Hickey and colleagues is not in all cases filtered in the same way. In Hickey et al. (2010a), the authors emphasize that there should be more interference by a singleton distractor when it is novel, meaning, in the current context, that it was not present on the preceding trial. Hitherto, we have filtered data according to the method described in that publication. In a later publication, Hickey et al. (2011) reversed this filter and limited their analyses to trials when a singleton distractor is present and was present on the previous trial. To make sure that we were not misrepresenting the comparison between the current study and those of Hickey et al. (2010a,b, 2011) we re-analyzed the data with the exact filters described in Hickey et al. (2011). In our four experiments, we did not find a single significant effect, whether main effects of color priming or previous rewards or an interaction between the two. The crucial interaction effect that constitutes reward priming had *p*-values ranging from.172 in experiment 1 to.799 in experiment 2. Even in experiment 4, where we presented the original stimulus materials of Hickey et al. (2010a,b, 2011), the effect vanished and the data did not show any reward priming. This is notable since we partially replicated the reward priming pattern in our initial analysis, with the filters based on Hickey et al. (2010a).

## General discussion

Hickey et al. (2010a,b, 2011) reported an intriguing effect of random reward, where attentional capture by an irrelevant singleton distractor decreased when the color scheme of the stimuli remained constant on consecutive trials, an effect they called reward priming. Conceptually identical paradigms tested here show that this effect is far from reliable—not generalizing well to other comparable situations. We do, however, partially replicate their result pattern in experiment 4 using their exact stimulus set, and a particular data filter. Hickey et al claimed that their findings reflected that primed features become more salient. This is consistent with what others have claimed for reward (e.g., Kristjánsson et al., 2010; Anderson et al., 2011). But the important difference here is that the rewards were not consistently related to a particular feature. In spite of the difficulties in replicating the reward priming effects using a different stimulus set, we acknowledge that it has been observed on three separate occasions (Hickey et al., 2010a,b, 2011), but our results cast doubt upon the simple salience-enhancement account of the effect. Why wouldn't the feature “green” become more salient, following reception of high reward, when presented as part of a Gabor-patch, when it does so as part of a circle or diamond shape? We have ruled out any role of set-size (experiment 2) and, at least, partially replicated the effect with a set-size of 4 (experiment 4). We have also shown that reward priming does not depend on whole-object processing and that our failures to replicate the effect in experiments 1 and 2 are unlikely to reflect insufficient motivation.

The discrepancy may potentially be explained by different task demands in the Gabor-patch version of the additional singleton paradigm, compared to the original. Might 1-trial reward priming depend on fast deployment of focal attention? Hickey and van Zoest (2012) showed that trajectories of saccadic eye-movements are strongly affected by the value of random reward on a preceding trial, and saccadic latencies. When latencies were short, performance was comparable to the visual search tasks revealing reward priming. Following high reward and a color swap between a target and a distractor, saccadic trajectories were “pulled” toward the distractor. Conversely, when previous reward was low and the color scheme was repeated, trajectories were repulsed away from the distractor. For long latencies, however, there was a repulsion effect for all conditions, as if the observers actively avoided the “pull” of the distractor. There may be an important difference in the deployment of attention in experiments 1–3 and experiment 4 here, in that to judge Gabor-patch orientation, focal processing may not be necessary, while in experiment 4, observers must focus on the target to discern the orientation of the central bar. A question for the future is therefore whether the reward priming effect is tied to the deployment of focal attention and, consequently, planning of saccades (although these may not necessarily be executed). Such an account is more nuanced than a simple salience enhancement account and a fully fleshed out version is beyond the scope of this article, but deserves attention in future.

For now, the most obvious conclusion to draw from our results, however, has to be that reward-color contingencies should be more consistent than here for reliable effects of reward upon attentional function. Kristjánsson et al. (2010) showed how the influence of reward, despite being flexible, builds up over time. It is therefore not unreasonable to expect that contingency between color and reward would induce stronger effects. It is, of course, difficult to define what a contingent relationship between reward and a feature really means in such a context. Even if stimuli are presented in random colors, the visual system probably regards coincidental sequences of repeated reward-color associations as something systematic. There must be a *first instance* of every possible association and, in that regard; a random presentation of reward and color is just a normal sequence of many such instances. It is unfortunate that the reward priming effect seems contingent on various co-occurrences (see the description of Data filtering in Experiment 1). Meaningful analysis of longer sequences, say up 3-trials back, therefore requires large amounts of data. It may be necessary to design experiments that bypass this problem to get a fuller picture of reward priming and how it develops over adjacent trials.

Another aspect of the design used by Hickey et al., which may merit further attention, is that while the main measure is response time, speed in responding is not the optimal task strategy. Motivating observers to respond quickly by limiting the time that they had for earning reward, did not affect the results however. This null-finding from one-experiment is of course far from being conclusive and the relationship of reward and strategy clearly merits further investigation (Shen and Chun, 2010; Della Libera et al., 2011). Della Libera et al. (2011) have argued for two separate reward-learning mechanisms in attention, one involving active monitoring of the contingencies, the other reflecting passive associations between reward and contingent stimuli (see also Camara et al., 2013). Clearly, only one of those can be operative under random reward schedules. Of great interest for future studies may be to uncover the conditions determining the operation of the two mechanisms.

## Conclusions

In sum, we are forced to the conclusion that reward priming is not a particularly robust effect, and cannot be fully accounted for by enhanced salience of rewarded features. Further studies will need to identify the boundary conditions of reward priming.

### Conflict of interest statement

The authors declare that the research was conducted in the absence of any commercial or financial relationships that could be construed as a potential conflict of interest.

## Footnotes

^1^For unspecified reasons, Hickey et al. (2011) apply the opposite filter, i.e., analyse only present trials succeeding present trials, when testing for the same reward priming effect (p. 122).

^2^To correct for the possiblity of unequal variances, we used Welch's t-test with corrected degrees of freedom.
